# 34 Self-inflicted Foreign Bodies in the Maxillary Sinus

**DOI:** 10.1016/S1808-8694(15)30162-2

**Published:** 2015-10-18

**Authors:** Márcio Meira Lima, Camila Alencar Moreira, Viviane Carvalho da Silva, Marcos Rabelo de Freitas

**Affiliations:** a MD, Otorhinolaryngologist at Hospital Geral do Exército de Fortaleza; b MD, Resident Physicial at the Otorhinolaryngology Service at Hospital Universitário Walter Cantídio - Universidade Federal do Ceará; c MSc in Public Health at Universidade Federal do Ceará, Assistant Physician in the Otorhinolaryngology Service at Hospital Universitário Walter Cantídio - Universidade Federal do Ceará; d PhD in Medical Sciences at Universidade Federal do Ceará, Assistant Professor in the Otorhinolaryngology Program at Universidade Federal do Ceará

**Keywords:** foreign body, maxillary sinus, sinusitis

## INTRODUCTION

Foreign bodies (FB) are a common occurrence in the realm of ENT practice. The most commonly involved sites are nasal cavities, ears, and pharynx. Foreign bodies are accompanied by site-related symptoms and do not pose much of a challenge to well-trained physicians. Foreign bodies may be introduced willingly by the patient or by accident^1^, ^2^.

Paranasal sinus foreign bodies are rarely seen, and most of them are introduced accidentally (25%) or iatrogenically (60%). The latter may occur as a consequence of dental, ophthalmic, and otorhinolaryngological procedures. The maxillary sinus is more frequently involved (75%), followed by the frontal sinus (18%)^3^, ^4^.

Only a few cases of self-inoculated paranasal sinus foreign body have been reported in the literature. This paper reports a case of chronic sinusitis secondary to the inoculation of multiple foreign bodies in the maxillary sinus.

## CASE REPORT

M.C., 49, sought ENT care complaining of nasal obstruction, halitosis, cacosmia, purulent rhinorrhea, and post-nasal drip for three years. Nasal fibroscopy showed a hypertrophied middle nasal concha to the right and a deviated septum (+++/4+) in Cottle”s area III convex to the left. The left middle meatus had a small number of polyps and thick purulent secretion. No signs of oroantral fistula were seen under oroscopy. The patient was diagnosed with chronic rhinosinusitis and was prescribed levofloxacin, 500mg/day orally for 21 days, prednisone, 40mg/day for 6 days and progressively lower dosages for up to 10 days, and nasal flushing with saline solution.

The patient improved only marginally. A CT scan of the paranasal sinuses was done after the patient followed the treatment described above. The maxillary sinuses were filled and the right middle concha was bullous. Functional endoscopic surgery was offered to the patient.

**Figure 1:**
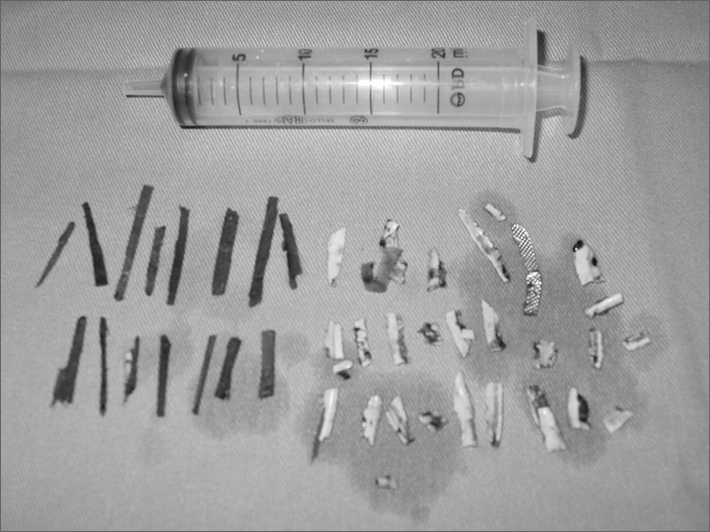
Foreign bodies removed from the left maxillary sinus

During left maxillary antrostomy multiple pieces of wood and plastic were found inside the sinus, and the operation had to be converted to an external approach using the Caldwell-Luc procedure to allow for better visualization of the antrum. Thirty-four foreign bodies were removed from the left maxillary sinus. Some of them broke into smaller pieces during surgical manipulation, and that is why on Figure 1 their number is larger. No foreign bodies were found on the right maxillary sinus. The day after surgery the patient revealed that he had undergone a dental extraction five years before, and that he began to voluntarily introduce foreign bodies in his sinus through an oroantral fistula. He showed no evident signs of mental disorder and could communicate normally. The patient improved dramatically from the symptoms, and post-nasal dripping persisted only for a few weeks after surgery.

## DISCUSSION

Foreign bodies in the paranasal sinuses are rare, but are an integral part of the differential diagnosis for rhinosinusitis, mainly when it occurs unilaterally. When symptoms appear at later stages, the most frequent complaints are indicative of chronic rhinosinusitis^5^.

The patient at hand had both maxillary sinuses involved. The foreign bodies precipitated the onset of left sinus disease. CT scans revealed bone failure on the floor of the left maxillary sinus, probably connected to the previously existing oroantral fistula. No foreign bodies were found on the right side, and the patient”s anatomic alteration (bullous middle concha) was probably the reason why this sinus was involved.

The Caldwell-Luc procedure combined with the endoscopic approach has been described as the golden standard for the treatment of various diseases of the maxillary sinus including foreign bodies, particularly when complete resolution cannot be achieved by functional endoscopic surgery^6^.

## CONCLUSION

Foreign bodies in the paranasal sinuses are rare. Oroantral fistulas are the most common inoculation path, mainly those secondary to dental procedures. The Caldwell-Luc procedure is the approach of choice to address this condition, mainly when it cannot be treated endoscopically.
